# Respiratory disorders associated with heavy inhalation exposure to dolomite dust

**Published:** 2012-09-30

**Authors:** M Neghab, R Abedini, A Soltanzadeh, A Iloon Kashkooli, S M A Ghayoomi

**Affiliations:** 1 PhD, Occupational Health Department, School of Health and Nutrition and Research Center for Health Sciences, Shiraz University of Medical Sciences, Shiraz, Iran; 2 MSc Student, Occupational Health Department, School of Health and Nutrition, Shiraz University of Medical Sciences, Shiraz, Iran; 3 MSc, Occupational Health Department, School of Health and Nutrition, Shiraz University of Medical Sciences, Shiraz, Iran; 4 Former MPh student, School of Health and Nutrition, Shiraz University of Medical Sciences, Shiraz, Iran; 5 Pneumologist, Associate professor, Department of Internal Medicine, School of Medicine, Shiraz University of Medical Sciences, Shiraz, Iran.

**Keywords:** Dolomite dust, Occupational exposure, Respiratory symptoms, Functional impairments of the lungs

## Abstract

**Background:**

Although dolomite is classified as a relatively non-toxic, nuisance dust, little information exists as to its potential to produce respiratory disorders following occupational exposure. The purpose of this study was, therefore, to evaluate the possible effects, if any, of heavy inhalation exposure to this chemical on the prevalence of respiratory symptoms, functional impairments and radiographic abnormalities of the lungs.

**Methods:**

The study population consisted of a group of 39 exposed subjects engaged in digging and excavating activities that were in operation for building a local dam, as well as 40 healthy non-exposed employees that served as the referent group. Subjects were interviewed and respiratory symptoms questionnaires, as suggested by the American Thoracic Society (ATS), were completed for them. Thereafter, they underwent chest X-ray and lung function tests. Additionally, using routine gravimetric techniques, personal dust monitoring for airborne inhalable and respirable dust was carried out at different dusty work sites. Finally to determine the chemical composition of the dust, it was analyzed by X-ray fluorescence (XRF) technique.

**Results:**

XRF revealed that the major component (50.52%) of the dust was calcium magnesium carbonate, dolomite. Additionally, levels of exposure to inhalable and respirable dust were estimated to be 51.7±24.31 and 23.0±18.11mg/m3, respectively. Statistical analysis of the data showed that symptoms such as regular cough, phlegm, wheezing, productive cough and shortness of breath were significantly (p<0.05) more prevalent among exposed workers. Similarly, the ratio of FEV1/FVC in exposed subjects was significantly different from that of non-exposed individuals. In contrast, no significant abnormalities were observed in the chest radiographs of both groups.

**Conclusions:**

In conclusion, while these data cast doubt on the notion that dolomite is a harmless chemical, they provide evidence in favour of the proposition that exposure to high atmospheric concentrations of this compound is likely to be associated with respiratory symptoms.

## Introduction

(1)Dolomite, calcium magnesium carbonate, with the chemical formula CaMg(CO3)2 is made up of 60% calcium carbonate (equivalent to 24% calcium) and 40% magnesium carbonate (equivalent to 12% magnesium). Déodat Gratet de Dolomieu, a French geologist, was the first to describe dolomite in 1791. He observed dolomite in a mountain in northern Italy, now named the Dolomite Alps. Dolomite rock (or dolostone) is mainly composed of the mineral dolomite(1)-(4).

In the past, given its chemical composition, dolomite used to be prescribed as a calcium and magnesium supplement. However, due to contamination with toxic elements such as lead and mercury, and availability of better supplementary forms of calcium and magnesium compound, it is no longer used as a therapeutic agent(4)-(6). No information exists to indicate that dolomite has any other therapeutic applications in human diseases (7)-(10).

Dolomite has also many industrial applications in construction, dam building, stone processing, chemical industries, asphalt, concrete and agriculture (soil fertilizer and pH control). Therefore large number of workers are occupationally exposed to dolomite, that may contain potentially toxic metals, including lead, arsenic, and mercury, which may lead to skin, blood, or neurologic disorders(10)-(14). Additionally, gastrointestinal (such as nausea and diarrhea)(15) and muscular problems (such as weakness)(4) have been reported in some subjects(16). Although the main occupational hazard in dolomite processing is dust and the chemical is classified as a relatively harmless, nuisance dust with a threshold limit value of 10 mg/m3 (ACGIH) or 15 mg/m3 (OSHA)(17), evidence for associations between exposure to dolomite dust and either respiratory symptoms or functional impairments of the lungs is lacking(15),(18).

To the best of the authors’ knowledge, to date, no systematic study has been carried out to assess the extent to which digging and excavating workers at the site of dam construction were exposed to dolomite dust. Additionally, no information exists as to the respiratory health of such workers following heavy inhalation exposure to this chemical. The present study, was, therefore, undertaken with the following objectives:

1.To assess the degree to which workers were exposed to dolomite dust.

2.To determine the prevalence of respiratory symptoms, if any, among dolomite workers as compared to a non-exposed referent population.

3.To find out if exposure to dolomite dust was associated with any acute or chronic significant decrements in the parameters of pulmonary function.

4.To determine if chest radiographs show any abnormal changes following exposure to dolomite dust.

## Materials and Methods

### Subjects:

This cross-sectional study was carried out at a construction site of a local dam in Fars province southern of Iran. All thirty nine male subjects who have had exposure to dolomite dust were investigated. Simultaneously, 40 healthy males with almost identical demographic and socioeconomic characteristics were selected by simple random sampling technique and served as the referent group. Both groups were volunteer subjects. The study was conducted in accordance with the Helsinki Declaration of 1964 as revised in 2000(19). All participants signed an informed consent form before commencement of the study. The prevalence of respiratory symptoms and changes in lung function values were studied in both groups. Additionally, in order to minimize the effects of confounding variables, workers with chronic respiratory diseases, asthma, allergies and history of lung infectious diseases excluded from the study.

### Measurements of study Variables

#### Respiratory Illness:

Subjects were interviewed and respiratory symptom questionnaire, as suggested by the American Thoracic Society (ATS, 1978)(20), with a few modifications, were administrated to all of them. This standardized questionnaire included questions on respiratory (presence or absence of regular dry and/or productive cough, wheezing, dyspnea, etc), nasal and eye symptoms, smoking habits, medical and family history of each subject. Additionally, it contained detailed occupational history and specific questions regarding all jobs held before employment at the plant under study, particularly those associated with the risk of respiratory morbidity. These, were then used to obtain symptom prevalence data among exposed and non-exposed groups.

### Measurement of Airborne Dust

To assess the extent to which workers had been exposed to dolomite dust, personal dust monitoring for airborne inhalable (particle size ≥5 µm) and respirable fractions (particle size <5 µm) was carried out at different dusty worksites. No standard sampling method exists for dolomite. Therefore, routine gravimetric sampling method used for silica dust, NIOSH silica sampling method (7601, issue 3, 2003), was used for this purpose(22). Application of this method is further justified when one considers the fact that dust contained small amounts of silica. To estimate the airborne dust concentration, a personal dust sampler (Casella, London-LTD), calibrated by a digital automatic calibrator connected to a filter holder equipped with a 25 mm membrane filter through which the air was aspirated by a battery-powered motor at a constant flow rate of 2 l/min was used(21). Based on a few preliminary tests, the optimum sampling time, to avoid overloading of the filters, was evaluated to be 2 hr. Dust concentration expressed in mg/m3 was calculated from the changes in the dried filter (for respirable fraction) or cyclone collector weight (for inhalable fraction), as measured by a digital scale at a sensitivity of 0.1 mg, before and after sampling, divided by the volume of air sampled.

### Determination of chemical contents of dust samples

To determine the exact chemical composition of the dust samples, they were analyzed by X-ray fluorescence (XRF) technique, on a fee for service basis, by a private analytical lab.

### Pulmonary Function Tests (PFTs)

Pulmonary function tests (PFTs), including mean percentage predicted vital capacity (VC), forced vital capacity (FVC), forced expiratory volume in the first second (FEV1 ) , peak expiratory flow (PEF), forced expiratory flow at 50% of the FVC (FEF 50 % ), followed guidelines given by the ATS (1979)(23) and measured with a portable calibrated vitalograph spirometer (Vitalograph- COMPACT, Buckingham-England) on site. The spirometer was calibrated twice a day with 1-liter syringe in accordance to the standard protocol for the instrument used. The mean percentage predicted value was based on subject age, body mass index (BMI), sex and ethnic group as calculated and adjusted by the spirometer device. Subjects were requested not to take shower or smoke for at least two hours prior to the test. Additionally, they were trained to become familiar with the maneuvers. The standing height and weight of each subject were measured in his normal working clothes. Before the test, they rested in a sitting position for about 5 min. They were then asked to stand in front of the spirometer, as comfortably as possible, and a nose clip was put on. At least, three acceptable maneuvers were performed. If subject showed great variability among the various FVC volumes, up to 5 maneuvers were obtained(24). The largest volumes (as percentage predicted lung function) were selected for analysis. The percentage predicted lung values were observed capacities as measured by a spirometer divided by predicted or expected capacities multiple by 100.

 % predicted lung value = (observed capacities/expected capacities) × 100.

Spirograms were evaluated and interpreted by the pneumologist author.

### Chest X-ray

Subjects were invited to a medical center and underwent Posterior-Anterior (PA) chest X-ray, using a Siemens instrument. Standard PA chest X-rays were read by the pneumologist author according to the ILO classification(25). The size of the film was 35×35 cm, the distance of the subjects from the X-ray tube was about six feet and the electrical voltage was 100 KV. 

### Data analysis 

The data were statistically analyzed using student’s t-test and chi-square or fisher's exact test, where applicable (with a preset probability of (P< 0.05). Experimental results are presented as arithmetic mean±SD. Association between lung function parameters and some variables such as duration of exposure was studied using multiple linear regression analysis. Statistical tests, using a two–sided P-value were conducted using SPSS software (version 11.5).

### Statistical procedures

Demographic variables of age and BMI as well as parameters of pulmonary function including VC, FVC, FEV1, PEF, FEV1/VC, FEV1/FVC and FEF 50% were compared between exposed and referent subjects by independent sample t-test. Conversely, statistical comparison of abnormalities in chest x-rays, prevalence of respiratory symptoms such as cough, phlegm, dyspnea, productive cough and wheezing as well as proportion of smokers in both groups were conducted by chi-square or fisher's exact test, where applicable. Association between length of exposure to dolomite dust and changes in the parameters of pulmonary function was evaluated by multiple linear regression analysis.

## Results

Demographic characteristics (such as age, BMI index and length of exposure), smoking habits and airborne concentrations of dolomite dust are shown in table 1. No significant differences were noted between exposed and non-exposed subjects as far as variables such as age, BMI, and smoking habits were concerned (p<0.05).

**Table 1:** Demographic characteristics, smoking habits of the study population and ambient air concentration of dolomite dust

parameter		Exposed (n=39)	Non-exposed (n=40)	P-value
**Age (year) (mean ± SD)**		38.41 ± 10.05	33.80 ± 8.09	0.925†
**Length of exposure (year) (mean ± SD) **		7.44 ± 2.47	N/A††	N/A††
**BMI (kg/m^2^) (mean ± SD)**		25.04±3.75	25.23±3.03	0.425†
**Respirable dolomite dust concentrations (mg/m3) (n=12)**		23.0±18.11	N/A††	-
**Inhalable dolomite dust concentrations (mg/m3) (n=12)**		51.7±24.31	N/A††	-
**Total dolomite dust concentrations (mg/m3) (n=12)**		74.7	N/A††	-
**Smoking habit**	**Smokers**	13 (33.3%)	11 (27.5%)	0.188‡
	**Non-smokers**	26 (66.7%)	29 (82.5%)	

^†^Independent sample t-test

^‡^Chi-square or fisher’s exact test

^††^Non-Applicable

Sample dust analysis, with X-ray fluorescence (XRF) technique, indicated that its major component (50.52%) was calcium magnesium carbonate (dolomite). Additionally, it contained 46.1% water, a small amount of SiO2 (1.61%) and other components including oxides of aluminum, iron, sodium, potassium, titanium and manganese.

Inhalable and respirable dust concentrations were estimated to be 51.7±24.31 and 23.0±18.11 mg/m3, respectively. Total dust concentration was estimated to be 74.7 mg/m3 (Table 1).

Table 2 illustrates the frequency of respiratory symptoms among exposed and non-exposed subjects. As shown, the prevalence of symptoms such as cough, phlegm, dyspnea, productive cough and wheezing in exposed subjects was significantly higher than in referent group (p<0.05). The parameters of pulmonary function were also measured for both groups. Predicted percentages of VC, FVC, FEV1, PEF, FEV1/VC, FEV1/FVC ratio and FEF 50% are presented in Table 3. As shown, all parameters of pulmonary function were lower for exposed subjects as compared with their non-exposed counterparts. However, only the FEV1/FVC ratio of exposed subjects was significantly different from referent subjects (p<0.05).

**Table 2:** Frequency of respiratory symptoms among exposed and non-exposed subjects

Symptoms	Exposed N (%) (N=39)	Non-exposed N (%) (N=40)	Odds ratio (CI95%)	P-value†
**Cough **	28(71.79)	4(10.0)	9.0 (2.7- 29.6)	0.0001*
**Phlegm **	29(74.35)	8(20.0)	6.25 (2.4-17.95)	0.0001*
**Dyspnea **	15(38.46)	1(2.5)	15.0 (1.98-113.51)	0.008*
**Productive cough**	19(48.71)	2(5.0)	18.0 (2.4-134.69)	0.004*
**Wheezing**	26(66.66)	3(7.5)	24.0 (3.24-177.4)	0.001*

^†^Chi-square or fisher’s exact test

^*^Significantly different from its corresponding value for the non-exposed group

**Table 3:** Predicted percentages of lung function among exposed and non-exposed subjects

Parameters	Exposed (n= 39)	Non-exposed (n= 40)	P-value†
**VC1**	82.18±12.91	84.50±10.06	0.148
**FVC2**	81.49±13.75	88.12±10.41	0.118
**FEV13**	83.46±13.05	84.40±13.15	0.628
**PEF4**	81.61±2193	86.15±18.02	0.220
**FEV1/VC**	102.03±10.20	99.91±10.83	0.937
**FEV1/FVC**	102.41±12.54	95.71±11.18	0.042*
**FEF 50%5**	71.66±22.40	76.15±22.86	0.928

^†^Independent sample t-test

^*^Significantly different from its corresponding value for the non-exposed group

^1^vital capacity (VC)

^2^forced vital capacity (FVC)

^3^forced expiratory volume in the first second (FEV1 )

^4^peak expiratory flow (PEF)

^5^forced expiratory flow at 50% of the FVC (FEF 50 % )

The results of chest radiographs are presented in Table 4. As shown, no significant differences observed in the chest radiographs of both groups Association between exposure to dolomite dust and changes in the parameters of pulmonary function was evaluated by multiple linear regression analysis (Table 5). The analysis showed that after adjusting for potential confounders, there was a significant association between length of exposure to dolomite dust and decrements in FEV1/VC and FEV1/FVC ratios (p<0.05).

**Table 4:** Frequency and percentages of lung radiographs abnormalities according to ILO classification among exposed and non-exposed subjects

Parameters	Exposed N (%) (n= 39)	Non exposed N (%) (n= 40)	P-value†
**Normal**	18 (46.2)	22 (55.0)	
**1.0**	12 (30.8)	9 (22.5)	
**1.1**	5 (12.5)	4 (10.0)	0.29
**2.1**	2 (5.1)	5 (12.5)	
**2.2**	2 (5.1)	0 (0.0)	

^†^Chi-square or fisher’s exact test

**Table 5:** Association between length of exposure to dolomite dust and changes in the parameters of pulmonary function

Parameters	Β-Coefficient	P-value†
**VC1**	0.245	0.134
**FVC2**	0.249	0.126
**FEV13**	-0.012	0.935
**PEF4**	-0.213	0.194
**FEV1/VC**	-0.398	0.012*
**FEV1/FVC**	0.331	0.039*
**FEF 50%5**	-0.254	0.119

^†^Multiple linear regression analysis

^*^Significantly different from its corresponding value for the non-exposed group

^1^vital capacity (VC)

^2^forced vital capacity (FVC)

^3^forced expiratory volume in the first second (FEV1 )

^4^peak expiratory flow (PEF)

^5^forced expiratory flow at 50% of the FVC (FEF 50 % )

## Discussion

According to the presented data, no significant differences exist between both groups as far as demographic variables such as age, BMI and smoking habits were concerned (Table 1). Additionally, none of the subjects had past medical or family history of respiratory illnesses or any other chest operations or injuries. Airborne concentration of dolomite dust was far beyond its current TLV of 10 mg/m3 (17). Therefore, it would be reasonable to attribute the prevalence of respiratory symptoms in exposed subjects to their exposure to dolomite dust and to tentatively conclude that exposure to this chemical in medium term (7.5 years) is likely to be associated with a significant increase in the prevalence of respiratory symptoms such as cough, phlegm, dyspnea, productive cough and wheezing.

Although, evidence for associations between exposure to dolomite dust and respiratory symptoms is limited(15)-(17), in some studies respiratory complaints such as cough and wheezing as a result of exposure to dolomite dust have been reported(18). The findings of the present study (Table 2) are in line with and further confirm these reports.

Furthermore, we have shown that exposure to dolomite dust resulted in small reductions in some parameters of pulmonary function such as VC, FVC, FEV1, PEF and FEF50%, although only the differences for FEV1/FVC ratio between exposed and referent subjects reached statistical significance. Therefore, one might conclude that, at least, under the scenarios described in this study, in terms of length and severity of exposure to dolomite dust, overt functional impairments of the lungs is not a major outcome. This conclusion is further supported by the fact that all parameters of pulmonary function in exposed subjects were in the normal range (≥80)(26).

Given the above, one might argue that the result of multiple linear regression analysis (Table 5) in which after adjusting for potential confounders, particularly, the important variable of age, significant associations observed between length of exposure and small decrements in FEV1/VC and FEV1/FVC ratios, do not support the aforementioned conclusion. While true, it has to be reiterated that long term consequence of heavy inhalation exposure to this chemical on lung functional capacities remains to be convincingly demonstrated by further studies.

In conclusion, the findings of this study collectively indicate that despite the fact that dolomite is classified as a relatively non toxic, nuisance dust, exposure to high concentrations of this chemical in medium term is likely to be associated with a significant increase in the prevalence of respiratory symptoms. Therefore, engineering measures such as local exhaust and dilution ventilation as well as the use of PPE are recommended to eliminate or reduce exposure to this chemical.

Additional studies with larger sample sizes and longer follow ups are clearly required to further confirm these initial observations and to ascertain whether ventilatory disorders and abnormal radiographic changes in the lungs are also outcomes of long term heavy inhalation exposure to dolomite dust.
